# A first-in-Asian phase 1 study to evaluate safety, pharmacokinetics and clinical activity of VS-6063, a focal adhesion kinase (FAK) inhibitor in Japanese patients with advanced solid tumors

**DOI:** 10.1007/s00280-016-3010-1

**Published:** 2016-03-30

**Authors:** Toshio Shimizu, Kazuya Fukuoka, Masayuki Takeda, Tutomu Iwasa, Takeshi Yoshida, Joanna Horobin, Mitchell Keegan, Lou Vaickus, Ajit Chavan, Mahesh Padval, Kazuhiko Nakagawa

**Affiliations:** Department of Medical Oncology, Kindai University Faculty of Medicine, 377-2 Ohno-higashi, Osaka-Sayama City, Osaka 5898511 Japan; Verastem, Inc., Needham, MA 02494 USA

**Keywords:** Focal adhesion kinase, Proline-rich tyrosine kinase-2, First-in-Asian phase 1 study, VS-6063, Defactinib

## Abstract

**Purpose:**

VS-6063 (also known as defactinib or PF-04554878) is a second-generation inhibitor of focal adhesion kinase and proline-rich tyrosine kinase-2. This phase 1 study evaluated the safety and tolerability, pharmacokinetics, and clinical activity of VS-6063 in Japanese subjects with advanced solid tumor malignancies in a first-in-Asian study setting.

**Methods:**

VS-6063 was administered orally twice daily (b.i.d.) in 21-day cycles to cohorts of three subjects each with a standard 3 + 3 dose-escalation design until disease progression or unacceptable toxicity. Blood samples for pharmacokinetics were collected on Day 1 and 15. The assessments were performed using CTCAE v4.0 for adverse events (AEs), and the Response Evaluation Criteria In Solid Tumors, version v1.1 (RECIST v1.1) for tumor response.

**Results:**

Nine patients were treated across three dose levels (200–600 mg BID). No dose-limiting toxicities were observed at any dose level. Most frequent treatment-related AEs were Grade 1/2 unconjugated hyperbilirubinemia, fatigue, decreased appetite, and diarrhea. Only one subject in the 200 mg BID cohort experienced reversible and transient Grade 3 unconjugated hyperbilirubinemia. PK analyses confirmed that the exposure at the recommended Phase 2 dose (RP2D) of 400 mg BID was comparable with exposures previously reported in non-Japanese subjects. Durable stable disease of approximately 24 weeks was confirmed in two subjects (malignant mesothelioma and rectal cancer).

**Conclusions:**

VS-6063 was well tolerated at all dose levels investigated in this first-in-Asian study. These data support the administration of VS-6063 to Japanese subjects at the RP2D in clinical trials involving solid tumor malignancies.

## Introduction

High expression of focal adhesion kinase (FAK) has been frequently demonstrated to be associated with invasive and metastatic malignancies, implicating FAK in malignant progression of multiple epithelial tumors [1–6]. FAK localizes to focal adhesions and mediates physical attachment of cells to the extracellular matrix (ECM). Focal adhesion kinase is known to promote tumor cell survival and resistance to anoikis (induction of apoptosis upon loss of contact with the ECM), via the ADRB2/Src signaling pathway. The ability to survive without ECM contact is a hallmark of metastatic cells, allowing them to leave the parent tumor and migrate to and colonize distant tissues. This ability is also characteristic of a subpopulation of highly tumorigenic cells which possess qualities like stem cell. These cells are considered to be cancer stem cells (CSC) or cancer or tumor initiating cells (CIC or TIC) and may be defined as malignant cells with a stem cell phenotype [7]. Tumor growth thus occurs by propagation of these cells, and CSC have been implicated in tumor initiation, progression, metastasis, and drug resistance. This mechanism of metastatic dissemination has recently been shown to involve the formation of filopodium-like protrusions and the subsequent establishment of elongated, mature adhesion plaques which contribute critically to the rapid proliferation of the micrometastatic cells within the new environment [8]. In these studies, activation of FAK was shown to be a critical protein involved in the proliferation of these cells. Taken together these data suggest that FAK modulation may be a potential therapeutic target for cancer.

VS-6063 (defactinib) is a small molecule, orally available potent adenosine 5′-triphosphate (ATP)-competitive, reversible inhibitor of FAK and proline-rich tyrosine kinase-2 (Pyk2). FAK and Pyk2 are members of the same family of nonreceptor protein tyrosine kinases sharing significant sequence homology and are implicated as important integrating molecules in signal transduction cascades. VS-6063 produces potent in vitro inhibition of recombinant human FAK and Pyk2 activity (inhibitory concentration of 50 % [IC50] = 0.6 nM for each kinase), displaying more than 100-fold greater selectivity for FAK and Pyk2 than for other nontarget kinases. Toxicology studies conducted with VS-6063 have identified the liver and gall bladder, gastrointestinal tract, hematopoietic system, testes/epididymis, and cardiovascular system as potential targets. Importantly, preclinical evidence indicates that VS-6063 has a low potential for CYP 2C-, 2D- or 3A-mediated drug interactions. Based on both these preclinical findings and previous first-in-human (FIH) experience of phase 1 study in subjects with advanced solid tumors in USA [9], a first-in-Asian phase 1 dose-escalation study was conducted to determine the MTD and overall safety and tolerability of VS-6063, and to identify a recommended phase 2 dose (RP2D) in Japanese patients with non-hematologic malignancies. Secondary objectives included characterization of the pharmacokinetic profile and documentation of preliminary antitumor activity.

## Materials and methods

### Patient eligibility

All subjects provided informed consent, and the study was conducted in accordance with both the Declaration of Helsinki and Good Clinical Practices (GCP), including the archiving of essential documents. The study was approved by the institutional review board of study site, and this study was registered at ClinicalTrials.gov as NCT01943292. The main eligibility criteria were as follows: Japanese patient with histologically or cytologically confirmed diagnosis of locally advanced or metastatic solid tumor malignancies that had experienced disease progression on, were intolerant of, or not eligible for standard therapy; aged 20 years or older; an Eastern Cooperative Oncology Group (ECOG) performance status of 0 or 1; and adequate hematologic, hepatic, and renal functions. Key exclusion criteria included cancer-directed therapy (chemotherapy, radiotherapy, hormonal therapy, biologic, or immunotherapy, etc.) within 28 days of the first dose of study drug or 5 half-lives, whichever was shorter, a gastrointestinal condition which could have interfered with the swallowing or absorption of study medication, corrected QT interval (QT_c_) ≥470 ms (as calculated by the Fridericia correction formula), uncontrolled or severe concurrent medical condition (including uncontrolled brain metastases), and uncontrolled or severe cardiovascular disease. Potent CYP3A4 inhibitors or inducers and systemic anticoagulation were prohibited.

### Study design and treatment

This was a phase 1, open-label, dose-escalation study to evaluate the safety and PK of VS-6063, a FAK inhibitor in Japanese subjects with advanced solid tumor malignancies. This study was expected to enroll up to 18 evaluable subjects with non-hematologic malignancies if 6 subjects were assigned at each of the 3 planned dose levels of 200, 400, and 600 mg BID. VS-6063 was administered orally, BID, in continuous 21 day cycles. Subject enrollment proceeded according to a standard 3 + 3 design. In the absence of DLT, each subject received oral VS-6063 (BID) for a minimum of 21 days of continuous daily dosing (1 cycle) and could continue to receive additional cycles of study treatment until disease progression was documented or other treatment discontinuation criteria were met. All patients within a cohort were required to complete 1 cycle (21 days) of dosing with <1 of 3 or <2 of 6 subjects experiencing a DLT prior to escalation to the next dose level in a new cohort. Three subjects were to be treated at each dose level until the first instance of DLT, after which up to 6 subjects were to be treated at that dose level. If a second DLT was observed in up to 6 subjects, the DLT dose level was reached. DLTs included Grade 4 neutropenia lasting ≥7 days without growth factor support; Grade ≥3 febrile neutropenia; Grade 4 thrombocytopenia, Grade 3 or 4 nonhematologic toxicities (excluding nausea, vomiting, or diarrhea unless uncontrolled with supportive care), Grade ≥3 QT_c_ interval prolongation, or any treatment-related toxicities that resulted in failure to receive at least approximately 85 % of the planned doses for that cycle (e.g., failure to complete at least 18 days of treatment in a continuous 21-day regimen) despite maximal (as judged by the investigator) supportive care measures. The MTD was defined as the highest dose level studied at which <1 subject out of 3 or <2 subjects out of 6 experienced a DLT. The RP2D was to be determined in discussion among the sponsor, medical monitor, and investigators. Observations related to PK and any VS-6063-related toxicities were included in the rationale supporting the RP2D. Safety and tolerability was assessed by the incidence and severity of AEs as determined by National Cancer Institute (NCI) Common Terminology Criteria for Adverse Events (CTCAE v4.03). The antitumor activity of study treatment was assessed according to the Response Evaluation Criteria in Solid Tumors (RECIST v1.1).

### Pharmacokinetic assessments

#### Blood sampling and processing

Pharmacokinetic samples were to be collected for determination of VS-6063 concentration and potential metabolites at the time points of Pre-dose (within 30 min of VS-6063 dosing), postdose (15 min, 30 min, and 1, 2, 4, 8, 12, and 24 h after VS-6063 administration) during Cycle 1 on Days 1 and 15. The analytical laboratory measured plasma concentrations using a validated method. Phoenix WinNonlin (v 6.3, Pharsight Inc., Mountain View, CA) was used to estimate all pharmacokinetic parameters using the non-compartmental analysis tool and the plasma model. *C*_max_ and *T*_max_ were determined by direct assessment (no calculations) of the concentration versus time data. All AUC calculations followed the linear trapezoidal rule. As data permitted, the terminal elimination rate constant (lambda *z*, *λz*) for VS-6063 data was calculated. Lambda *z* was determined by the slope of the regression line of the natural log transformed concentrations versus time with at least three data points not including the *C*_max_ and a correlation coefficient (*R*^2^) of regression >0.90. In instances where the *λz* range was not at least twofold greater than the calculated half-life, the resulting values (AUCINF, CL/*F* and *V*_z_/*F*) were deemed unreliable and were not reported, although the half-life will be calculated and reported if the *λz* range met the *R*^2^ requirements. The following PK parameters were to be calculated: oral clearance (CL/*F*), maximum concentration (*C*_max_), *T*_max_, half-life (T1/2), area under the curve (AUC), and other relevant parameters.

#### Urine collection and sampling

To assess the elimination of VS-6063 and its potential metabolites, total 24-h urine output was to be collected on Cycle 1 Day 1 and Cycle 1 Day 15 in conjunction with PK sampling.

## Results

### Patient characteristics

A total of 9 subjects received VS-6063 between September 2013 and June 2014. All were Asian (of Japanese descent). The study population was predominantly male (77 %), with an ECOG performance status of 0 or 1 (100 %). The most common cancer type was rectal cancer (3 patients). Other cancer types, including esophageal, lung, mesothelioma, Paget’s carcinoma, colon, and thymic cancer, were each reported in 1 subject. Eight subjects overall had received prior systemic therapy for their cancer, with a median of 7 prior therapies across dose cohorts. Six subjects, 2 in each dose cohort, had prior surgery. Only 1 subject, in the 200 mg cohort, had received prior radiation therapy (Table 1).

**Table 1:** Baseline characteristics of the patients (*n* = 9)

Characteristics	All patients, *n* = 9
Age (years)	
Median	60.4
Range	38.0–75.0
Sex	
Male	7
Female	2
ECOG performance status	
0	7
1	2
Primary tumor	
Colorectal cancer	4
Esophageal cancer	1
Mesothelioma	1
NSCLC	1
Thymic cancer	1
Extramammary Paget’s disease	1
No. of prior systemic therapy	
Median	7
Range	0–13

*ECOG* Eastern Cooperative Oncology Group

### Safety and tolerability

Treatment-related adverse events (AEs) occurring in at least two subjects are summarized in Table 2. The most commonly reported AEs overall were unconjugated hyperbilirubinemia (7 patients, 78 %), fatigue (6 patients, 67 %), decreased appetite (4 patients, 44 %), and diarrhea (3 patients, 33 %). Only one patient in the 200 mg dose cohort experienced a Grade 3 AE of unconjugated hyperbilirubinemia. All other toxicities were manageable and were predominantly mild in intensity (Grade 1 or Grade 2) in severity. There were no AEs leading to death or SAEs, and no AEs leading to early study withdrawal. No DLTs were reported in any dose cohort. Hyperbilirubinemia was asymptomatic and its onset typically occurred within the first 2 weeks of initiating treatment. Patients with Grade 1 or 2 unconjugated hyperbilirubinemia were able to continue dosing, although bilirubin levels tended to fluctuate during treatment. Hyperbilirubinemia was reported across all dose cohorts, for 3 (100 %) patients in the 200 mg dose cohort, 2 (67 %) patients in the 400 mg dose cohort, and 2 (67 %) patients in the 600 mg dose cohort. One event of hyperbilirubinemia (200 mg cohort) was Grade 3 in severity. This patient had Grade 1–2 hyperbilirubinemia starting on Day 7; the Grade 3 event began on Day 42 and resolved 6 days after onset following interruption of study drug. All reports of hyperbilirubinemia were considered to be related to defactinib. None of these subjects had concurrent increases in ALT or AST above ULN. The most common events of gastrointestinal disorders were diarrhea reported in 3 (33 %) subjects and nausea reported in 2 (22 %). Diarrhea was reported in 1 (33 %) subject in the 400 mg dose cohort and 2 (67 %) subjects in the 600 mg dose cohort. Nausea was reported in 1 (33 %) subject in the 200 mg dose cohort, and 1 (33 %) subject in the 600 mg dose cohort. Both reports of nausea were mild in severity as were 2 of the 3 reports of diarrhea; 1 subject in the 600 mg group experienced diarrhea of moderate intensity. No clinically meaningful changes in any ECG parameter were observed for any dose cohort and no subject had a QT_c_ interval ≥500 ms or QT_c_ increase from baseline >30 ms.

**Table 2:** Treatment-related adverse events occurring in at least 2 subjects

	VS-6063 dose cohort			Total *N* = 9 *n* (%)
	200 mg BID *N* = 3 *n* (%)	400 mg BID *N* = 3 *n* (%)	600 mg BID *N* = 3 *n* (%)	
Blood bilirubin increased	3 (100.0)	2 (66.7)	2 (66.7)	7 (77.8)
Fatigue	2 (66.7)	1 (33.3)	3 (100.0)	6 (66.7)
Decreased appetite	2 (66.7)	1 (33.3)	1 (33.3)	4 (44.4)
Diarrhea	0	1 (33.3)	2 (66.7)	3 (33.3)
Aspartate aminotransferase increased	1 (33.3)	0	1 (33.3)	2 (22.2)
Blood alkaline phosphatase increased	2 (66.7)	0	0	2 (22.2)
Nausea	1 (33.3)	0	1 (33.3)	2 (22.2)
Headache	0	1 (33.3)	1 (33.3)	2 (22.2)
Anemia	0	0	2 (66.7)	2 (22.2)

### Pharmacokinetics

**Fig. 1:**
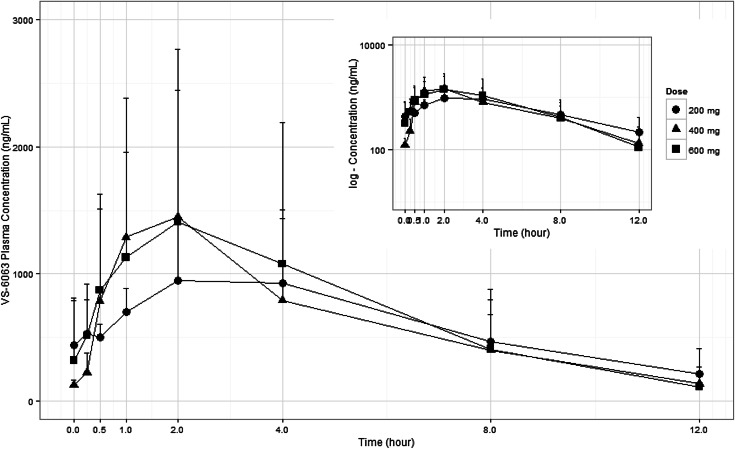
Steady-state serum VS-6063 concentration–time profiles over the 12-h dose interval (VS-6063 doses 200–600 mg twice daily). PK analysis of previous first-in-human phase 1 study revealed that VS-6063 dose of 163 mg twice daily (per 70-kg person) is projected to achieve a steady-state average plasma concentration equivalent to the predicted efficacious free (non-protein-bound) plasma concentration of 13.3 ng/mL required to produce 50 % inhibition of tumor pFAK activity in humans

VS-6063 was rapidly absorbed, with median *T*_max_ observed at 2.0 h (range 0.5–4.0 h) postdose following oral doses of 200–600 mg BID. Plasma VS-6063 exposure (*C*_max_ and AUC) increased in a less than dose proportional manner and the mean AUC0-12 and AUC0-24 values remained relatively consistent across the full dose range evaluated (Fig. 1). Doses above 400 mg BID did not result in a significant increase in VS-6063 exposure. A similar *C*_max_ was observed in the 400 and 600 mg BID dose cohorts on both Days 1 and 15 and mean AUC values were relatively consistent across the 200–600 mg BID dose range. Clearance appeared to be increased with dose on both Days 1 and 15, which is consistent with the less than dose proportional increase in exposure. Median half-life values ranged from 2.3 to 4.3 h across all dose regimens and both PK study days. The Day 1 mean CL/F values were 45.6, 105, and 204 L/h for the 200, 400, and 600 mg doses, respectively. The Day 15 mean CL/*F* values were 32. 1, 70, and 123 L/h for the 200, 400, and 600 mg doses, respectively (Table 3). VS-6063 was detected in the urine of all patients and appeared consistent across all dose cohorts. The mean renal clearance (CLr) values ranged from 0.0855 to 0.179 L/h, and the percent relative to the total dose administered ranged from 0.0439 to 0.356 %. All 9 subjects had systemic concentrations of the 4 metabolites of VS-6063 that were evaluated (M2, M3, M4, and M5). Median plasma *T*_max_ values for all metabolites were observed at 2.0–4.0 h postdose administration for all cohorts on both PK sampling days. Based on the relative plasma *C*_max_ and AUC0-12 values for the metabolites compared to VS-6063 values, M2 was the most abundant metabolite, followed by M4, M3, and then M5. Both the M2 and M4 exposures appeared to be >10 % of the parent exposure, while M3 and M5 had exposures that were <10 % of the parent exposure. In the urine, the M2 metabolite was the most abundant and was excreted in amounts greater than the parent compound.

**Table 3:** Serum pharmacokinetic parameters of VS-6063 in Japanese patients with advanced solid tumors on Day 1 and Day 15

Dose (mg)	Day	*C*_max_, µg/mL GM (CV %)	*T*_max_, h, median (range)	AUC_0–12h_, µg h/mL GM (CV %)	AUC_0-∞_, µg h/mL GM (CV %)	*t*_1/2_, h, median (range)	*V*_z_, L GM (CV%)	CL/F (L/h) GM (CV%)
200 mg BID *n* = 3	Day 1	0.63 (0.11)	2 (0.5, 4)	3.78 (153)	4.39 (175)	4.3 (3.5, 5.5)	288 (144)	45.6 (175)
	Day 15	0.93 (55)	2 (1, 4)	6.24 (90)	NA	2.95 (2.9, 3.0)	334, 99.6*	32.1 (90)
400 mg BID *n* = 3	Day 1	0.67 (333)	2 (2, 4)	3.37 (159)	3.84 (131)	2.45 (2.3, 3.5)	410 (189)	105 (133)
	Day 15	0.96 (218)	2 (1, 4)	5.71 (136)	NA	3.0 (2.89, 3.11)	174 (63.5)	70 (136)
600 mg BID *n* = 3	Day 1	0.61 (319)	2 (1, 2)	2.68 (269)	3.0 (232)	2.79 (1.33, 5.24)	790 (576)	204 (242)
	Day 15	0.98 (216)	2 (2, 4)	4.88 (322)	NA	2.27 (1.58, 2.95)	158, 3060*	123 (322)

AUC_0–12h_, area under the concentration–time curve from time zero to 12 h (dosing interval); AUC_0–∞_, are under the concentration–time curve from time zero to infinity; *C*_max_, maximum observed serum concentration; CV, coefficient of variation; PK, pharmacokinetic; *t*1/2 elimination half-life; *T*_max_, time of maximum observed serum concentration; GM, Geometric mean

* *N* = 2; therefore, individual values are presented

### Antitumor activity

The best response overall was stable disease for 3 (33 %) out of 9 subjects, 1 subject in each dose cohort. Remarkably, durable stable diseases by RECIST criteria were maintained for 169 days (24 weeks) in a subject with mesothelioma in the 400 mg cohort, and for 162 days (23 weeks) in a subject with rectal cancer in the 600 mg dose cohort. The overall median time to disease progression for all subjects was 63 days (9 weeks) and ranged from 61 to 64 days across dose cohorts.

## Discussion

This was a first-in-Asian phase 1, open-label, dose-escalation study to evaluate the safety and PK of VS-6063 in subjects of Japanese descent with non-hematologic malignancies. The overall purpose of this phase 1 study was to support the development of VS-6063 as an oral formulation for the treatment of Asian (Japanese) subjects with advanced solid tumors. The doses and regimen proposed for this study were based on both nonclinical data and findings of the FIH phase 1 study. The rationale for a BID schedule was based on nonclinical data supporting the requirement for prolonged suppression of the target for maximal efficacy and the observed human half-life of 9.0 h. In the previous FIH phase 1 study, subjects received doses of 12.5–750 mg of defactinib BID for continuous 21 day cycles. PK exposure of VS-6063 at the RP2D of 400 mg BID was consistent with previously reported data in non-Japanese patients. Doses above 400 mg BID did not result in a significant increase in VS-6063 exposure. Available data indicate that 400 mg BID should result in adequate plasma concentration levels to suppress FAK activity.

Following the first-generation FAK inhibitor drug development including PF-00562271 [10], early drug development of several potent selective second-generation FAK inhibitors has been progressed recently. In the phase 1 dose-escalation study of another second-generation FAK inhibitor BI 853520, both proteinuria and fatigue were dose-limiting (Grade 3) at the higher doses [11, 12]. In this study, no subject developed any grade of proteinuria; nevertheless, the most common AE in the general disorders was fatigue reported in 6 (67 %) subjects. Fatigue was reported across all dose cohorts with 2 (67 %) subjects in the 200 mg dose cohort, 1 (33 %) subject in the 400 mg dose cohort, and 3 (100 %) subjects in the 600 mg dose cohort. One subject in the 400 mg dose cohort had an interruption in study drug for 13 days because of moderate fatigue beginning on Day 78. All other reports of fatigue were mild in severity. Regarding safety, increased bilirubin was the most commonly reported laboratory abnormality across all dose cohorts. Most patients had at least one Grade 1 or Grade 2 unconjugated hyperbilirubinemia. Transient increases in total bilirubin were most pronounced at C1D8; however, values for all affected subjects trended toward normal range by the end of the study. In only 1 of the 9 subjects, a dose modification was required for increased bilirubin. The increases observed in blood bilirubin did not appear to be dose related, and were transient and/or sporadic. There was no increase in ALT or AST values associated with the increases in bilirubin. The current hypothesis for occurrence of unconjugated hyperbilirubinemia with VS-6063 is that either the parent compound and/or its metabolite compete with bilirubin for hepatic conjugation and elimination. Of note, both VS-6063 and its metabolites are UGT1A1 inhibitors in vitro. VS-6063 is metabolized in the liver by oxidative metabolism and its metabolite undergoes glucuronide conjugation via uridine diphosphate glucuronosyltransferase (UGT) 1A1, the hepatic enzyme primarily responsible for conjugation of bilirubin [13, 14].

In pharmacokinetics analysis, VS-6063 was rapidly absorbed, with median *T*_max_ observed at 2 h postdose following oral doses of 200–600 mg BID. Plasma VS-6063 exposure (*C*_max_ and AUC) was less than dose proportional and doses above 400 mg BID did not result in a significant increase in VS-6063 exposure. A similar *C*_max_ was observed in the 400 and 600 mg BID dose cohorts on both Days 1 and 15, and mean AUC values were relatively consistent across the 200–600 mg BID dose range. Clearance appeared to increase with dose on both Days 1 and 15, which is consistent with the less than dose proportional increase in exposure. Exposure at 400 mg BID (RP2D) was comparable to that previously reported in non-Japanese patients [9].

The best overall response in this study was stable disease, reported in 3 of the 9 patients, including 1 patient in each dose cohort. Durable stable disease of approximately 24 weeks was observed in 1 subject with mesothelioma and 1 subject with rectal cancer in the 400 and 600 mg dose cohorts, respectively. No objective radiographic responses were observed with VS-6063, which is similar to findings with the first-generation compound PF-00562271 [10] and another second-generation FAK inhibitors currently in development, GSK2256098 [15] and BI 853520 [11, 12]. Inhibition of FAK prevents tumor invasion and dissemination rather than reducing tumor bulk. FAK inhibition is not overtly cytotoxic and is expected to be most effective when used in combination with other chemotherapeutic drugs. Recent studies have suggested specific patient populations that may benefit from FAK inhibition. For example, in a signaling network involving extracellular signal-related kinase (ERK), RhoA and FAK are dysregulated in high-grade non-small cell lung cancer (NSCLC) occurring against a background of Ink4a-Arf deficiency [16]. Suppression of RhoA or FAK selectively induced death in mutant Kras/Ink4a-Arf–deficient NSCLC cells, and pharmacologic inhibition of FAK caused regression of high-grade NSCLC in mutant Kras/Cdkn2a-null mice [16]. Based on these data, a study of VS-6063 has been initiated in patients with NSCLC harboring a Kras mutation (ClinicalTrials.gov, NCT01951690). In addition, recent preclinical data revealed that FAK promotes anti-tumor immune evasion. Specifically, the kinase activity of nuclear-targeted FAK in squamous cell carcinoma (SCC) cells drives exhaustion of CD8 + T cells and recruitment of regulatory T cells (Tregs) in the tumor microenvironment by regulating chemokine/cytokine and ligand–receptor networks, including via transcription of Ccl5, which is crucial. A small molecule FAK kinase inhibitor, VS-4718, which is currently in clinical development, also drives depletion of Tregs and promotes a CD8 + T cell-mediated anti-tumor response. Therefore, FAK inhibitors may trigger immune-mediated tumor regression, providing previously unrecognized therapeutic opportunities [17]. These findings could be a strong rationale for combination strategy with FAK inhibitors and immune checkpoint inhibitors including anti-PD-1 and anti-PD-L1 antibodies.

In summary, VS-6063 has an acceptable safety profile with Grade 1–2 adverse events that are easily managed and reversible, even with continued dosing, in Japanese subjects with advanced solid tumor malignancies. PK analyses confirmed that the exposure at the RP2D of 400 mg BID was comparable with that previously reported in non-Japanese subjects. Data from this study support VS-6063 administration to Japanese subjects with advanced solid tumor malignancies in investigational trials. The favorable safety profile of VS-6063 creates the opportunity to combine this drug with other agents or to test it as monotherapy in the adjuvant setting. For use as monotherapy, it will be important to identify suitable candidate populations [18–21].
